# Intermittent Exposure to a Single Bottle of Ethanol Modulates Stress Sensitivity: Impact of Age at Exposure Initiation

**DOI:** 10.3390/cells12151991

**Published:** 2023-08-03

**Authors:** Paige Marsland, Sarah Trapp, Andrew Vore, Ashley Lutzke, Elena I. Varlinskaya, Terrence Deak

**Affiliations:** Behavioral Neuroscience Program, Department of Psychology, Binghamton University, Binghamton, NY 13902-6000, USA

**Keywords:** alcohol, neuroimmune, adolescent intermittent ethanol, lipopolysaccharide, drinking, rat model

## Abstract

Alcohol use during adolescence is a serious public health problem, with binge drinking and high-intensity drinking being particularly harmful to the developing adolescent brain. To investigate the adverse consequences of binge drinking and high-intensity adolescent drinking, adolescent rodents were intermittently exposed to ethanol through intragastric gavage, intraperitoneal injection, or vapor inhalation. These models revealed the long-lasting behavioral and neural consequences of adolescent intermittent ethanol (AIE) exposure. The present study was designed to characterize a different AIE model, namely, intermittent exposure to a single bottle of 10% ethanol as the only source of fluids on a 2 days on/2 days off (water days) schedule, and to determine whether this AIE exposure model would produce changes in hormonal and neuroimmune responsiveness to challenges of differing modalities. Assessments of ethanol intake as well as blood and brain ethanol concentrations (BECs and BrECs, respectively) in adult male and female rats (Experiment 1) revealed that BECs and BrECs peaked following access to ethanol for a 2 h period when assessed 1 h into the dark cycle. Experiment 2 revealed age differences in ethanol intake, BECs, and BrECs following a 2 h access to ethanol (1 h into the dark cycle), with adolescents ingesting more ethanol and reaching higher BECs as well as BrECs than adults. In Experiment 3, intermittent exposure to a single bottle of 10% ethanol for 10 cycles of 2 days on/2 days off was initiated either in early or late adolescence, followed by an acute systemic immune challenge with lipopolysaccharide (LPS) in adulthood. LPS increased corticosterone and progesterone levels regardless of sex and prior ethanol history, whereas an LPS-induced increase in cytokine gene expression in the hippocampus was evident only in ethanol-exposed males and females, with females who underwent early exposure to ethanol being more affected than their later-exposed counterparts. In Experiment 4, intermittent ethanol exposure in females was initiated either in adolescence or adulthood and lasted for 12 ethanol exposure cycles. Then, behavioral (freezing behavior), hormonal (corticosterone and progesterone levels), and neuroimmune (cytokine gene expression in the PVN, amygdala, and hippocampus) responses to novel environments (mild stressors) and shock (intense stressors) were assessed. More pronounced behavioral and hormonal changes, as well as changes in cytokine gene expression, were evident in the shock condition than following placement in the novel environment, with prior history of ethanol exposure not playing a substantial role. Interleukin (IL)-1β gene expression was enhanced by shock in the PVN, whereas shock-induced increases in IL-6 gene expression were evident in the hippocampus. Together, these findings demonstrate that our intermittent adolescent exposure model enhances responsiveness to immune but not stress challenges, with females being more vulnerable to this AIE effect than males.

## 1. Key Findings

- The present experiments revealed age differences in ethanol intake during exposure to a single bottle of 10% ethanol solution as the only liquid available, with adolescent rats ingesting more ethanol than adults and demonstrating higher blood and brain ethanol concentrations.
- Chronic intermittent ethanol exposure using a single bottle of 10% ethanol solution as the only liquid available that was initiated either in early or late adolescence enhanced cytokine expression to lipopolysaccharide (LPS) challenge, with this effect being more evident in females following early ethanol exposure.
- Chronic intermittent ethanol exposure of females to a single bottle of 10% ethanol initiated in adulthood exacerbated behavioral signs of distress (freezing), an effect that was not evident after adolescent ethanol consumption.

## 2. Introduction

Adolescent drinking is a serious public health problem in the United States and worldwide. According to the Substance Abuse and Mental Health Services Administration [1], 1.8 million adolescents aged 12 to 17 initiated alcohol use in 2021 [1], whereas in 2022, 52% of high school seniors used alcohol within the last 12 months [2]. Early initiation of alcohol use is viewed as a risk factor for developing alcohol use disorder (AUD) later in life. Adolescents who begin drinking before the age of 15 are more likely to develop AUD than those who begin to use alcohol later in adolescence [3,4]. While adolescents tend to drink less frequently than adults, they typically consume higher quantities of alcohol per occasion, often demonstrating binge drinking, defined as the consumption of at least five drinks by males or four drinks by females within a 2 h period that brings blood alcohol concentrations to 80 mg/dL or higher [5]. Some adolescents also report high-intensity drinking, characterized by consuming 10 or more drinks per occasion [6]. Binge drinking and high-intensity drinking are thought to be particularly harmful to the developing adolescent brain, and findings from human longitudinal studies suggest that these patterns of alcohol use are associated with neural alterations and cognitive impairment [7,8]. Substantial maturational changes occur in the brain during adolescence, including reductions in the volume of gray matter via synaptic pruning, as well as concurrent increases in white matter volume associated with continued myelination of axons [9]. Adolescent binge drinking and heavy drinking can affect these developmental trajectories, with accelerated gray matter volume decreases and attenuated white matter volume increases evident in drinking adolescents relative to their non-drinking counterparts [10]. Adolescents with a history of binge drinking demonstrate elevations in risky decision-making and impulsive behavior, as well as impairments in executive functions, attention, and memory [7,9,11]. There is some evidence that cognitive alterations associated with adolescent binge drinking are more pronounced in females than males [12]. Neurobehavioral consequences of adolescent alcohol use may be related, at least in part, to alcohol-induced neuroinflammation, since numerous preclinical studies have shown that alcohol activates immune signaling in the brain [13,14].

Laboratory rodent models of adolescence have been extensively used for the identification of adverse consequences associated with adolescent binge drinking [7,15,16,17]. Employment of laboratory rodent models in alcohol research is based on certain similarities between human adolescents and adolescent rats and mice in terms of behavioral, hormonal, and neural alterations that occur during this developmental period [18]. Behaviorally, adolescent rats, like human adolescents, find interactions with peers more rewarding than adult rats, demonstrate increased levels of novelty-seeking as well as risk-taking, and voluntarily consume more ethanol per occasion than adults [18,19,20]. Furthermore, region- and system-specific neural alterations that occur during adolescence and contribute to adolescent-typical behavioral alterations are conserved across mammalian species [18,19]. In rats, the age range between postnatal day (P) 25 and P45 is considered the pre- and peri-pubertal period of early to mid-adolescence, whereas the age range between P45 and P65 is viewed as late adolescence/emerging adulthood [21,22].

Although no currently available animal model can reproduce complex human behaviors, including alcohol use, some findings from preclinical studies are consistent with human research. For instance, adolescent intermittent ethanol (AIE) exposure in laboratory rodents produces long-lasting cognitive deficits, with tasks that require behavioral flexibility (i.e., the ability to change behavior in response to changing environmental contingencies) being particularly affected [23,24,25]. Other behavioral alterations associated with AIE exposure include increases in anxiety-like behavior, impulsivity, and risk taking [25,26,27,28,29]. Similar to humans, laboratory rodents with a history of AIE exposure show structural brain alterations, including increased volumes of the orbitofrontal cortex and cerebellum [30]. Importantly, animal models of adolescent alcohol exposure not only replicate human data but also provide insight into new areas of research. Recent animal studies have found AIE-associated long-lasting alterations in neuroimmune function [14,31,32]. These neuroimmune alterations include activation of microglia [33,34], upregulation of multiple pro-inflammatory Toll-like receptors (TLRs) and nuclear factor kappa B (NFκB) signaling [29], as well as enhanced expression of cytokines and chemokines in the brain [33,35,36]. Cytokine expression induced by acute ethanol challenge is also enhanced in adult rats with a history of AIE [35].

Recent preclinical studies of AIE-associated consequences that included both sexes have shown sex differences in the induction of pro-inflammatory markers [37]. For instance, AIE-exposed female mice demonstrated increased levels of several cytokines and chemokines in the PFC during acute withdrawal, with no such changes evident in males [38]. Adult female rats with a history of AIE but not their male counterparts showed enhanced expression of interleukin (IL)-1β in the hippocampus [35]. Together, these findings suggest that females may be more sensitive to AIE-associated alterations in neuroimmune function, although more studies that include both sexes are needed for a better understanding of the sex-dependent consequences of AIE exposure.

For the investigation of adverse consequences associated with binge drinking and high-intensity drinking during adolescence, adolescent rodents have been intermittently exposed to ethanol through intragastric gavage (IG), intraperitoneal injection (IP), or vapor inhalation [15,35,39]. In these exposure paradigms, adolescent rats or mice receive relatively high doses of ethanol, resulting in rapid elevation of blood ethanol concentration (BEC) as well as brain ethanol concentration (BrEC). These AIE models have certain advantages. For instance, IG ethanol administration allows for precise control over the dose of ethanol given to the animal, and ethanol absorption occurs through the gastrointestinal tract. IP ethanol administration results in very rapid absorption and high peak BECs. Exposure to ethanol vapor is a non-invasive procedure that allows BECs to be kept at a targeted level for hours. However, all these AIE exposure models have some limitations. AIE exposure to 4–5 g/kg ethanol via IG gavage requires administration of large volumes into the stomach, with ethanol absorption dependent on the gastric content at the time of administration [40]. The main limitation of IP ethanol administration is that ethanol injected directly into the peritoneal cavity can cause irritation as well as peritoneal inflammation [41]. In general, experimental subjects are exposed to ethanol vapor for 14 h [42], and this duration of intoxication seems too long for modeling adolescent binge drinking [43].

While adolescent rats demonstrate higher ethanol intake than adult rats, most rat strains avoid ethanol and are not inclined to consume unadulterated ethanol solutions, making it difficult to develop a voluntary consumption-based approach for determining long-lasting neurobehavioral alterations associated with AIE exposure. However, rats will ingest ethanol when it is the only source of fluids (i.e., the forced ethanol consumption paradigm). Chronic consumption of ethanol as the only liquid available brings BECs to 95–100 mg/dL, with adolescents consuming more ethanol per kg of body weight and demonstrating higher BECs than adults [44]. Intermittency of chronic ethanol exposure during adolescence, mimicking human adolescent drinking patterns, may be necessary for inducing long-lasting behavioral and neural alterations [45]. We have shown previously that chronic intermittent ethanol exposure to a single bottle providing ethanol is sufficient to induce amyloid beta deposits in aging rats (10–14 months of age), which was also associated with an apparent increase in phagocytic activity of microglia, a common index of neuroimmune priming [46].

The main goal of the present experiments was to characterize adolescent intermittent exposure to a single bottle of 10% ethanol as the only source of fluids on a 2 days on/2 days off (water days) schedule and determine whether this exposure paradigm would produce changes in hormonal and neuroimmune responsiveness to challenges of differing modalities. For better characterization of the model, in Experiment 1, we assessed intake from a single bottle of 10% ethanol in the home cage, as well as corresponding BECs and BrECs in adult males and females. Experiment 2 was designed to test whether ethanol intake as well as BECs and BrECs would differ in adolescent and adult rats. Our previous studies have shown that AIE exposure via intragastric gavage produced long-lasting and sex-specific changes in stress sensitivity [47] and neuroimmune responses [35,36]. Therefore, Experiment 3 was designed to test the generalizability of these AIE-induced alterations using intermittent exposure to a single bottle of 10% ethanol for 2 days, followed by water for 2 days, instead of intragastric gavage. Given previous studies that have observed timing-specific effects of AIE, with more pronounced consequences evident after exposure during early to mid-adolescence than late adolescence/emerging adulthood [21], intermittent exposure to the single ethanol bottle was initiated either during early or late adolescence and was followed by an acute systemic immune challenge with lipopolysaccharide (LPS) in adulthood. Following Experiment 3, we found that females appeared to be uniquely responsive to the age of initiation of ethanol exposure. In Experiment 4, we followed up on this finding by using the intermittent exposure to a single bottle of ethanol of female rats initiated either in early adolescence or adulthood and followed by a stress challenge. Animals were either placed in a novel environment (moderate intensity stressor) or exposed to shock (high-intensity stressor), and their behavioral, hormonal, and neuroimmune stress responses were assessed.

## 3. Materials and Methods

### 3.1. General Methods

#### 3.1.1. Subjects

Experimental subjects were adolescent and adult male and female Sprague–Dawley rats bred on site from breeders purchased from Envigo. Litters were culled on postnatal day (P) 1 to 8–10 pups, with an even sex ratio maintained (4–5 males and 4–5 females). On P21, rats were weaned and pair-housed with a same-sex, non-littermate in standard Plexiglas cages with chew blocks provided for enrichment. Animals were pair housed in a temperature-controlled (22 ± 1 °C) vivarium maintained on a 12:12 h light/dark cycle (lights on at 0700) with ad libitum access to food prior to and for the duration of all experiments (Purina Rat Chow, Lowell, MA, USA). Rats were handled for 2–3 min for 2 days prior to all experimentation. Animals were maintained and treated in accordance with PHS policy and the Institutional Care and Use Committee (IACUC) at Binghamton University, and experimental protocols were approved prior to procedures.

#### 3.1.2. A Single Bottle Chronic Intermittent Ethanol Exposure Paradigm

In this study, experimental subjects were chronically exposed to a single bottle of ethanol on a 2 days on/2 days off (water days) schedule. Ethanol-consuming groups were given 48 h access to a single bottle of 10% (*v*/*v*) ethanol solution as the only source of fluids, followed by 48 h access to a single bottle of tap water [46]. This 4-day cycle was repeated 10 times (Experiment 3) for a total of 40 days, or 12 times (Experiment 4) for a total of 48 days. Bottle weights were recorded immediately prior to drinking, 24 h following access, and immediately following the drinking cycle to approximate the amount of ethanol consumed. Body weights were assessed prior to ethanol exposure for each 4-day cycle. Bottles were changed and weighed approximately 1 h prior to the lights going off to minimize disruption to the heavy bout of food and fluid consumption that occurs in rats and mice in the first few hours of the dark cycle.

### 3.2. LPS Challenge

LPS purchased from Sigma (serotype E0111:B4) was diluted in sterile, pyrogen-free physiological saline (0.9%) to a 0.1% concentration (1.0 mg/mL) and stored at −20 °C. On the day of experimentation, LPS solution was prepared fresh to a 0.001% concentration (100 µg/mL) and given IP in a volume of 1.0 mL/kg.

### 3.3. Stress Challenge

In Experiment 4, females underwent a procedure of varying stress intensity to examine the effects of chronic intermittent exposure to a single ethanol bottle on behavioral fear responses indexed via freezing behavior [48]. Rats were assigned to one of three conditions: (1) being allowed to stay in their home cage (HC) until the time of tissue collection; (2) being placed in a novel environment for 30 min (NE); or (3) receiving 3 electric shocks in a novel environment (SH). Both the novel environment as well as shock conditions took place in Med Associates footshock, sound-attenuating chamber (model MED-VFC-SCT-R; Med Associates Inc., Fairfax, VT, USA). In the NE condition, animals were placed in the footshock chambers (no shocks were delivered), and behavior was recorded with a near-infrared camera and scored using automated Med Associates freezing software. Subjects were recorded at 30 frames per second, and an instance of freezing was defined as no movement for at least 18 frames. In the SH condition, animals were placed in the chambers for a 5 min acclimation period followed by three presentations of scrambled footshock (3 shocks, 1.0 mA, 1 s each, variable intertrial interval = 90 s) delivered over the course of 3 min, shock parameters adapted from [48]. Animals were left in the chambers for another 22 min (a total of 30 min in the shock environment). Subjects were recorded, and freezing behavior was scored as defined above. In both NE and SH conditions, tissue collection occurred at the end of the 30 min period. Chambers were cleaned after each session with a wet paper towel.

### 3.4. Tissue Collection

Non-anesthetized rats were rapidly decapitated, and trunk blood collected into EDTA-coated glass collection tubes was centrifuged to separate plasma. Plasma was stored at −20 °C until protein assay analysis. In Experiments 1 and 2, brain regions were collected by gross dissection and stored at −80 °C until analysis. For Experiments 3 and 4, brains were removed, flash frozen in 2-methylbutane on dry ice, and then stored at −80 °C until punched on a Leica cryostat. Tissue punches were taken using Paxinos and Watson’s 2nd Edition Brain Atlas from the paraventricular nucleus of the hypothalamus (PVN), amygdala (AMG), ventral hippocampus (vHPC), and dorsal hippocampus (dHPC). Tissue punches were stored in 2 mL microcentrifuge tubes at −80 °C for PCR analysis.

### 3.5. Corticosterone and Progesterone Analysis

Corticosterone was assessed in plasma using a commercially available EIA kit (ADI-901-097; Enzo Life Sciences, Farmingdale, NY, USA). Samples were heat-inactivated to denature endogenous corticosteroid binding globulin (CBG) in 75 °C water for 60 min, and then the manufacturer’s instructions were followed. Inter-assay variability was 2.3%, and intra-assay variability was 1.7%. Progesterone was assessed similarly in plasma using a commercially available EIA kit (ADI-901-011; Enzo Life Sciences, Farmingdale, NY, USA). Samples were heat inactivated and denatured in 75 °C water for 60 min, and the manufacturer’s instructions were then followed. Inter-assay variability was 3.7%, and intra-assay variability was 1.0%.

### 3.6. Reverse-Transcriptase Polymerase Chain Reaction

RT-PCR was conducted using procedures described previously [49]. Reagent (Invitrogen, Grand Island, NY, USA) was spiked into tissue punches along with a 5 mm stainless-steel bead, then homogenized using a Qiagen Tissue Lyser (Qiagen, Valencia, CA, USA). RNA was extracted using RNeasy mini columns (Qiagen) and eluted in 65 °C RNase-free water. Nanodrop (ThermoScientific, Waltham, MA, USA) was used to determine RNA concentration and quality, and then normalized using RNase-free water. A QuantiTect reverse transcription kit (Qiagen) was used to synthesize cDNA following the manufacturer’s instructions. cDNA was amplified in a 10 μL reaction, consisting of 0.5 μL cDNA, 5 μL SYBR Green Supermix (Bio-Rad, Hercules, CA, USA), 0.5 μL primer, and 4 μL RNase-free water. RT-PCR was conducted using a CFX384 real-time PCR detection system (Bio-Rad) in a 384-well plate. Samples were pipetted in triplicate and underwent a 3 min start, then were denatured at 95 °C for 30 s, annealed (30 s at 60 °C), and extended (30 s at 72 °C) for a total of 40 cycles. To ensure product alignment, samples were again denatured for 1 min at 95° C and annealed for 1 min at 55 °C. Following annealing, samples were heated at a rate of 0.5 °C every 15 s until 95 °C to analyze the melt curve for specificity to the target gene. Data were analyzed relative to expression of the ultimate control using the 2^ΔΔC(t)^ method [50].

### 3.7. Blood and Brain Ethanol Concentrations

BECs and BrECs were measured by head-space gas chromatography using a Clarus 580 Gas Chromatograph. For BrECs, tissue samples were weighed, and 2 mL/g of water was added to each sample. A 5 mm stainless-steel bead was added to each tube and homogenized using a Qiagen Tissue Lyser. A 25 μL aliquot of each sample was placed into an airtight vial and loaded into the headspace. BECs and BrECs were determined using TotalChrom software by comparing the peak under the curve of each sample to the standard curve. BECs were recorded in mg/dL and BrECs were recorded in mg/100 g brain tissue.

### 3.8. Statistical Analysis

Data were analyzed using Prism software and Statistica to perform factorial ANOVA with the designs described below. Significant main effects and interactions were further clarified using Tukey’s HSD where appropriate.

### 3.9. Experimental Designs and Procedures

**Experiment** **1:** *Blood and brain ethanol concentrations in adult male and female rats following exposure to a single bottle of 10% ethanol for 2, 4, or 6 h*.

**Figure 1:**
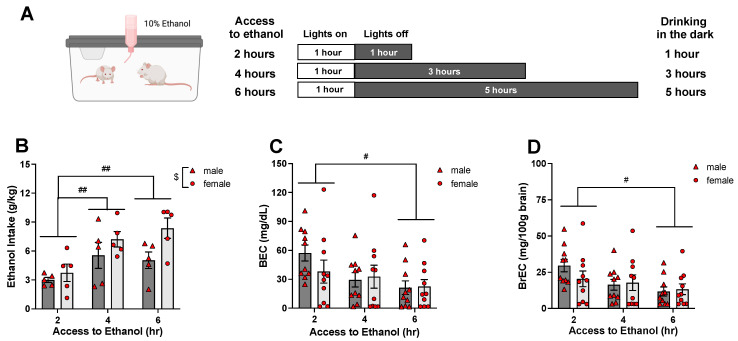
Experiment 1: Timeline (**A**), ethanol intake, and blood and brain ethanol concentrations (BEC and BrEC, respectively). N = 60, n = 10/group. Ethanol intake (**B**) was significantly higher in adult female rats than in their male counterparts, with data collapsed across time of ethanol access. Animals given access to ethanol for 2 h (1 h into the dark cycle) ingested significantly less ethanol than those given access for 4 or 6 h. BECs (**C**) and BrECs (**D**) were the highest in animals given access to ethanol for 2 h and differed significantly from the BECs and BrECs of animals given access to ethanol for 6 h. $ marks sex differences, *p* < 0.05; # marks differences between ethanol exposure timing, with data collapsed across sex, *p* < 0.05, ##—*p* < 0.01.

Experiment 1 assessed peak blood and brain ethanol concentrations following access to a single bottle of 10% ethanol [23]. In this experiment, adult (P75-83) male and female Sprague–Dawley rats were given access to a single bottle of 10% ethanol solution for 24 h on Day 1. On Day 2, animals were given access to ethanol for 2, 4, or 6 h (1, 3, or 5 h into the dark cycle), and blood and brain samples were collected (see Figure 1A for details). BrECs were assessed in the hippocampal tissue. Ethanol intake on a g/kg basis was estimated by subtracting initial bottle weights from final bottle weights, then dividing by the sum of animal body weights within the cage. Ethanol intakes on Day 1 (24 h access) and Day 2 (2, 4, or 6 h access) were measured and analyzed. The design of Experiment 1 was a 2 (sex) × 3 (time of drinking in the dark) factorial, with 10 animals placed in each group (N = 60).

**Experiment** **2:** *Blood and brain ethanol concentrations in adolescent and adult rats following exposure to a single bottle of 10% ethanol for 2 h*.

It has been shown previously that adolescent rats consume more ethanol and consequently reach higher BECs than adults [44,51,52]. Therefore, Experiment 2 was designed to investigate age-related drinking patterns from a single bottle of 10% ethanol by assessing ethanol intake, BECs, and BrECs. Adolescent (P28-31) and adult (P81-87) male and female rats were given access to a single bottle of 10% ethanol for 24 h on Day 1. On Day 2, animals were given 2 h access to ethanol (1 h before and 1 h after lights were turned off) and euthanized for blood and brain tissue collection. Ethanol intake on Day 1 and Day 2, as well as blood and brain ethanol concentrations, were assessed and analyzed. The design of this experiment was a 2 (age) × 2 (sex) factorial, with 10 animals placed in each of the four groups (N = 40).

**Experiment** **3:** *Intermittent exposure to a single bottle of ethanol followed by the LPS challenge: Initiation of exposure in early or late adolescence*.

The timing of AIE exposure may play a substantial role in persistent alterations evident later in life, with ethanol exposures initiated during early to mid-adolescence often producing more behavioral and neural alterations than those beginning later in adolescence [21]. To examine whether initiation of adolescent intermittent ethanol exposure to a single bottle of 10% ethanol in early (P28) versus late (P48) adolescence will alter responsiveness to acute systemic immune challenge with bacterial lipopolysaccharide (LPS), adolescent male and female rats underwent the single bottle of 10% ethanol drinking procedure for a total of 10 cycles. The control animals received a single bottle of water. In order to minimize experimenter handling, ethanol exposure was not calculated. Following cessation of drinking, animals underwent an abstinence period of 7–10 days to fully abate withdrawal from ethanol in adulthood. Animals were then challenged with 100 µg/mL LPS given IP, with blood tissue collected 3 h post-injection. Plasma stress hormones (corticosterone and progesterone) as well as gene expression of cytokines in the hippocampus were assessed, as previous work from our lab has indicated that the hippocampus is highly sensitive to neuroimmune gene-related changes following AIE [35]. There were four groups in this experiment: water-exposed challenged with a vehicle, water-exposed challenged with LPS, exposed to ethanol in early adolescence challenged with LPS, and exposed to ethanol in late adolescence challenged with LPS. LPS-induced changes in corticosterone, progesterone, and cytokine gene expression were assessed relative to water-exposed controls challenged with a vehicle. Genes of interest include c-Fos, IL-6, IL-1β, TNFα, and IkBα. c-Fos was chosen as a reporter of neuronal and glial activity in the area [53]. IL-6, IL-1β, and TNFα are cytokines that are upregulated following chronic ethanol in adulthood [54] and, along with IkBα, are responsive to LPS exposure [49]. The effects of ethanol exposure on LPS responsiveness were assessed relative to water-exposed controls challenged with LPS. The design of Experiment 3 was a 2 (sex) × 4 (exposure/challenge group) factorial, with 10 animals placed in each of the eight groups (N = 80).

**Experiment** **4:** *Intermittent exposure to a single bottle of ethanol of adolescent and adult females followed by varying the stressor challenge*.

Results from Experiment 3 indicated that females were uniquely sensitive to ethanol exposure in adolescence, so males were not included in this experiment. To determine whether a history of chronic intermittent exposure to a single bottle of 10% ethanol during adolescence or adulthood would modulate responsiveness to a moderate and intense stressor in females, we compared the behavioral, hormonal, and immune responses to a novel environment and footshock (see Stress Challenge). Intermittent ethanol exposure was initiated in adolescence (P28-32) or adulthood (P91-95) and 12 ethanol exposure cycles were followed by 7–10 days of abstinence from ethanol. In order to assess the amount of ethanol consumed within a cage, bottle weights were taken daily and animal body weights were taken once per cycle in order to estimate g/kg and mL/kg of ethanol or water consumed, respectively. Then, ethanol-exposed females and naïve to ethanol water-drinking controls underwent the varied stress challenge in adulthood (P85-87 for adolescent exposure and P140-145 for adult exposure), as described above. Animals were euthanized following the stress challenge (~30 min after stress onset) or at an equivalent time for nonstressed controls, with blood and brain tissue collected for later analysis. Plasma corticosterone levels as well as c-Fos, IL-1β, IL6, and CCL2 gene expression were assessed in the PVN, AMG, and HPC. These regions of interest were targeted based on their involvement in stress reactivity, contextual fear conditioning, and negative affect regulation more broadly.

The design of this experiment was a 2 (age of initiation: adolescent, adult) × 2 (exposure: water, ethanol) × 3 (stressor challenge: HC, NE, SH) factorial, with 10 animals placed in each adolescent and adult home cage group, and 12 adult females placed in each SH stress condition (N = 124).

## 4. Results

**Experiment** **1:** *Blood and brain ethanol concentrations in adult male and female rats following exposure to a single bottle of 10% ethanol for 2, 4, or 6 h*.

Although Day 1 ethanol intake was comparable among groups (see Table 1), *F*(1, 24) = 0.532, *p* = 0.5944, it differed as a function of sex, *F* (1, 24) = 7.249, *p* < 0.05, with females consuming more ethanol than males (13.85 ± 1.58 and 7.52 ± 1.28 g/kg, respectively). The ANOVA of ethanol intake on Day 2 revealed the main effects of sex, *F*(1, 24) = 6.32, *p* < 0.05, and time, *F*(3, 24) = 7.83, *p* < 0.01. As on Day 1, females consumed significantly more ethanol than males (6.44 ± 0.72 g/kg versus 4.54 ± 0.58 g/kg). Ethanol intake was significantly lower following a 2 h access period than following both 4 h (*p* < 0.01) and 5 h (*p* < 0.01) access periods (see Figure 1B).

The ANOVA of BECs revealed a main effect of time, *F*(2, 54) = 4.009, *p* < 0.05, with significantly (*p* < 0.05) higher BECs evident following ethanol access for 2 h than for 6 h (Figure 1C). Similarly, BrECs in the hippocampus differed as a function of time, *F*(2, 54) = 4.214, *p* < 0.05, with BrECs significantly (*p* < 0.05) higher following 2 h compared to 6 h of access to a single bottle of 10% ethanol (Figure 1D).

**Experiment** **2:** *Blood and brain ethanol concentrations in adolescent and adult rats following exposure to a single bottle of 10% ethanol for 2 h*.

**Figure 2:**
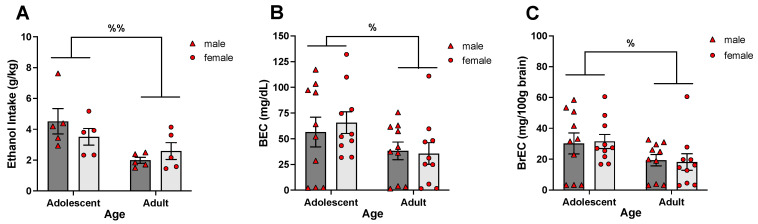
Experiment 2: Ethanol intake, BEC, and BrEC in adolescent and adult male and female rats. N = 40, n = 10/group. Ethanol intake (**A**) from a single bottle of 10% ethanol during a 2 h access was significantly higher in adolescents than adults, with adolescents also demonstrating significantly higher BECs (**B**) and BrECs (**C**) relative to their adult counterparts. Age differences are marked with %—*p* < 0.05 and %%—*p* < 0.01.

Experiment 2 was designed to test the impact of age on ethanol intake from a single bottle as well as BECs and BrECs in the hippocampus achieved following a 2 h access. Ethanol intake on Day 1 (see Table 1) differed as a function of age, *F*(1, 16) = 21.93, *p* < 0.001, with adolescents ingesting more ethanol than adults (14.03 ± 1.45 g/kg versus 6.60 ± 0.62 g/kg). However, the ANOVA of ethanol intake on Day 1 revealed no main effect of sex. Age differences in ethanol intake were evident on Day 2 as well, *F*(1, 16) = 9.18, *p* < 0.01, with adolescents consuming more ethanol than adults (Figure 2A). No main effect of sex was observed, with males and females consuming comparable amounts of ethanol.

The ANOVA of BECs revealed a main effect of age, *F*(1, 36) = 4.63, *p* < 0.05, with adolescents reaching higher BECs than adults (Figure 2B). BrECs also differed as a function of age, *F*(1, 36) = 5.43, *p* < 0.05, with higher BrECs evident in adolescents than adults (Figure 2C). No main effects of sex were evident for both measures, with males and females achieving similar BECs and BrECs.

**Experiment** **3:** *Intermittent exposure to a single bottle of ethanol followed by the LPS challenge: Initiation of exposure in early or late adolescence*.

Body weights were assessed separately in males and females during both early and late exposure using 2 (exposure) x 10 (cycle) ANOVA. In males, gradual body weight increases across cycles were evident during early and late exposures, as evidenced by significant main effects of cycle, *F*(9, 162) = 4865, *p* < 0.0001, and *F*(9, 162) = 293.4, *p* < 0.0001. However, male body weights were not affected by exposure to a single bottle providing ethanol during either early, *F*(1, 18) = 2.17, *p* = 0.158, or late adolescent exposure, *F*(1, 18) = 0.489, *p* = 0.493 (Supplemental Figure S1A,B). In female subjects, two-way ANOVA also revealed significant main effects of cycle, *F*(9, 162) = 1772, *p* < 0.0001, and *F*(9, 162) = 293.4, *p* < 0.0001, with gradual body weight increases across cycles evident during both early and late adolescent exposures, *F*(9, 162) = 4865, *p* < 0.0001, and *F*(9, 144) = 153.2, *p* < 0.0001. In females, body weights were not affected by intermittent drinking from a single bottle providing ethanol during either early, *F*(1, 18) = 0.021, *p* = 0.886, or late adolescence, *F*(1, 12) = 0.271, *p* = 0.610 (Supplemental Figure S1C,D).

**Figure 3:**
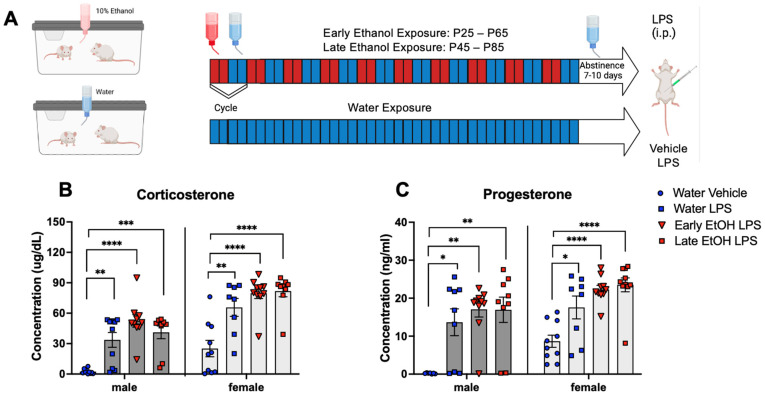
Experiment 3: Timeline (**A**), corticosterone, and progesterone. N = 80, n = 10/group. Plasma corticosterone (**B**) as well as progesterone (**C**) levels were significantly elevated by LPS relative to water-exposed controls challenged with saline in both males and females. Significant LPS-induced changes are marked with *—*p* < 0.05, **—*p* < 0.01, ***—*p* < 0.001, ****—*p* < 0.0001.

Plasma stress hormones (corticosterone and progesterone) and gene expression of cytokines in the hippocampus were assessed separately in males and females using one-way ANOVA. As expected, baseline measures of both corticosterone and progesterone were very low in males [55]. In both males and females, corticosterone levels were affected by LPS, *F*(3, 35) = 14.47, *p* < 0.0001 and *F*(3, 33) = 15.01, *p* < 0.001, respectively, with all LPS-challenged groups demonstrating significant corticosterone and progesterone increases relative to same sex controls exposed to water and challenged with a vehicle (Figure 3B,C).

**Figure 4:**
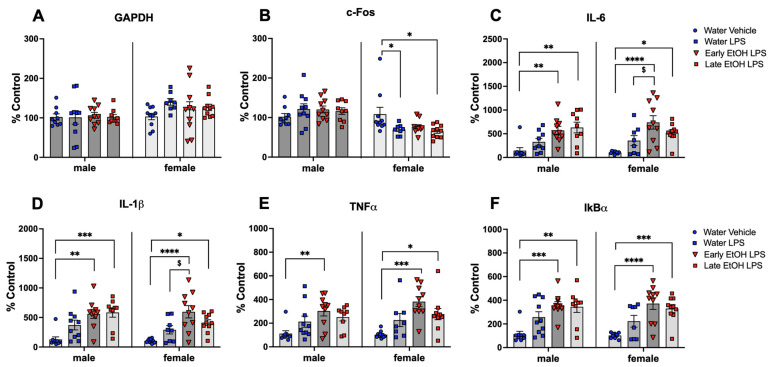
Experiment 3: Gene expression in the hippocampus. N = 80, n = 10/group. GAPDH (**A**) gene expression was not affected by LPS in both males and females. Expression of c-Fos (**B**) was not affected by LPS in males, whereas LPS significantly decreased c-Fos gene expression in water-exposed females as well as in females exposed to ethanol during late adolescence relative to water-exposed controls injected with a vehicle. IL-6 (**C**) as well as IL-1β (**D**) gene expression was significantly increased by LPS in males and females with a history of early and late ethanol exposure. Females exposed to ethanol during early adolescence demonstrated significantly higher LPS-induced IL-6 and IL-1β expression than their water-exposed counterparts challenged with LPS. TNFα gene expression (**E**) was enhanced by LPS only in the early exposed to ethanol males, while females exposed to ethanol during early or late adolescence showed significant LPS-induced increases in TNFα. IκBα gene expression (**F**) was significantly increased by LPS in males and females with a history of both early and late ethanol exposure. Significant LPS-induced changes relative to water-exposed controls injected with a vehicle are marked with *—*p* < 0.05, **—*p* < 0.01, and ***—*p* < 0.001, ****—*p* < 0.0001. Significant differences between water-exposed and ethanol-exposed subjects challenged with LPS are marked with $—*p* < 0.05.

GAPDH expression was not affected by LPS in both males, *F*(3, 34) = 0.047, *p* = 0.986, and females, *F*(3, 34) = 1.448, *p* = 0.246 (see Figure 4A). Expression of c-Fos was not affected by LPS in males, *F*(3, 34) = 0.071, *p* = 0.519, whereas in females, the one-way ANOVA revealed significant differences among experimental conditions, *F*(3, 34) = 3.821, *p* < 0.05, with LPS significantly decreasing c-Fos expression in water-exposed females as well as in females exposed to ethanol during late adolescence relative to water-exposed controls injected with a vehicle (Figure 4B). In both males and females, adolescent exposure to ethanol, regardless of exposure timing, resulted in enhancement of IL-6 expression induced by LPS, *F*(3, 33) = 6.958, *p* < 0.001 and *F*(3, 34) = 8.827, *p* < 0.001, respectively (Figure 4C). While water-exposed males and females did not show increases in IL-6 expression following LPS challenge, both ethanol exposure conditions demonstrated significant increases in IL-6 relative to water-exposed controls challenged with a vehicle. Furthermore, females exposed to ethanol early demonstrated significantly higher LPS-induced IL-6 expression than their water-exposed counterparts. Similar patterns of IL-1β expression were evident in both males, *F*(1, 34) = 8.074, *p* < 0.001, and females, *F*(3, 34) = 9.701, *p* < 0.0001 (see Figure 4D). Animals of both sexes exposed to ethanol either during early or late adolescence demonstrated significant increases in IL-1β gene expression relative to water-exposed controls challenged with a vehicle, whereas water-exposed males and females did not show significant increases in IL-1β expression following LPS challenge. Females who were exposed to ethanol early also demonstrated significantly higher IL-1β expression relative to water-exposed controls challenged with LPS. In males, expression of TNFα was affected by LPS only in the early exposed to ethanol group, *F*(3, 34) = 4.363, *p* < 0.05, with only this group demonstrating significantly higher TNFα gene expression relative to water-exposed control males challenged with a vehicle (see Figure 4E). In females, LPS challenge affected expression of TNFα, *F*(3, 34) = 8.349, *p* < 0.001, with females in both ethanol-exposure conditions demonstrating significant LPS-induced increases in TNFα relative to water-exposed controls injected with a vehicle. IκBα expression also differed among groups in males, *F*(3, 34) = 8.28, *p* < 0.0, and females, *F*(3, 34) = 10.61, *p* < 0.0, with significant LPS-induced increases relative to water-exposed controls injected with a vehicle evident in animals following early and late ethanol exposure, but not in their water-exposed counterparts challenged with LPS (see Figure 4F).

**Experiment** **4:** *Intermittent exposure to a single bottle of ethanol by adolescent and adult females followed by varying the stressor challenge*.

Body weight changes across cycles were assessed in adolescent and adult females using a separate 2 (exposure) × 12 (cycle) ANOVA. These analyses revealed main effects of cycle for adolescent and adult exposures, *F*(11, 638) = 30146, *p* < 0.0001, and *F*(11, 682) = 36.13, *p* < 0.0001, respectively, with gradual body weight increases across cycles evident at both ages. However, body weights were not affected by exposure to a single bottle providing ethanol during adolescent, *F*(1, 58) = 2.602, *p* = 0.112, or adult exposure, *F*(1, 62) = 2.623, *p* = 0.110 (See Table 2, Supplemental Figure S1E,F).

**Figure 5:**
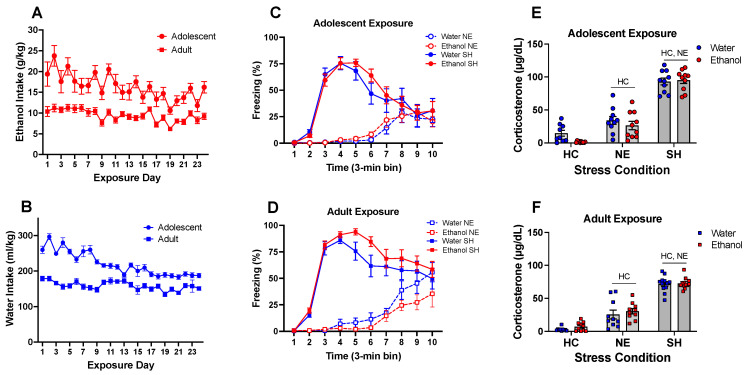
Experiment 4: Ethanol intake, water intake, unconditioned freezing, and corticosterone. In general, adolescent females ingested significantly more ethanol than adults (**A**); however, this age difference did not reach statistical significance on Days 6, 12, 15, 16, 19, 20, and 23. Adolescent females ingested significantly more water on multiple days (**B**), although age differences were not evident on Days 13, 18, 22, and 23. Adolescent exposure to ethanol, however, did not affect freezing behavior (**C**). Females in the SH group demonstrated significantly more freezing behavior than animals placed in a novel environment, with significant differences between stressors evident during time bins 3, 4, 5, 6, and 7. Exposure to ethanol in adulthood did not affect freezing behavior either. Females in the SH condition showed significantly more freezing than their NE counterparts during time bins 3, 4, 5, 6, 7, and 8 (**D**). In females exposed to ethanol or water during adolescence, animals placed in the NE demonstrated significantly higher corticosterone levels than the HC control, and the SH group had significantly higher corticosterone levels than the other two groups (**E**). In females exposed to ethanol or water during adulthood, females in the NE stress condition had significantly higher corticosterone levels than the HC control, and animals in the SH group demonstrated greater corticosterone levels than females in both the HC and NE stress conditions (**F**). Significant changes relative to the home cage control are marked with HC—*p* < 0.05, while significant changes relative to the novel environment stress condition are marked with NE—*p* < 0.05, with data collapsed across exposure.

The ANOVA of ethanol intake across the drinking cycles revealed significant main effects of day, *F*(23, 689) = 6.571, *p* < 0.0001, and age, *F*(1, 30) = 56.93, *p* < 0.0001, as well as day by age interaction, *F*(23, 689) = 1.708, *p* < 0.05. In general, adolescent females ingested significantly more ethanol than adults; however, this age difference did not reach statistical significance on Days 6, 12, 15, 16, 19, 20, and 23 (see Figure 5A). Intake of adolescents averaged across drinking cycles was 16.34 ± 0.63 g/kg per 24 h, while adults ingested on average 9.34 ± 0.28 g/kg of ethanol. The ANOVA of water intake measured on the first two days of each cycle in water controls also revealed main effects of day, *F*(23, 667) = 17.05, *p* < 0.0001, age, *F*(1, 29) = 86.30, *p* < 0.0001, as well as day by age interaction, *F*(23, 667) = 9.548, *p* < 0.0001 (see Figure 5B). Adolescent females ingested significantly more water on multiple days, although age differences were not evident on Days 13, 18, 22, and 23. Given these age differences in ethanol and water intake, further analyses of chronic intermittent ethanol exposure effects on stress responsiveness were separated by age.

Following adolescent exposure, the ANOVA of freezing behavior revealed significant main effects of stressor, *F*(1, 36) = 59.02, *p* < 0.01 and time, *F*(9, 324) = 28.45, *p* < 0.0001, as well as stress by time interaction, *F*(9, 324) = 35.67, *p* < 0.0001. Initiation of ethanol exposure during adolescence, however, did not affect freezing behavior, with exposure by stress interaction not reaching statistical significance, *F*(1, 36) = 0.001, *p* = 0.972. As expected, animals in the SH group demonstrated significantly more freezing behavior than animals placed in the novel environment, with significant differences between stressors evident during time bins 3, 4, 5, 6, and 7 (see Figure 5C). Following adult exposure, the ANOVA of freezing also revealed significant main effects of stressor, *F*(1, 40) = 117.6, *p* < 0.0001, and time, *F*(9, 360) = 47.46, *p* < 0.0001, as well as a significant stressor–time interaction, *F*(9, 360) = 38.75, *p* < 0.0001. Exposure to ethanol in adulthood did not affect freezing behavior, with exposure by stress interaction not reaching statistical significance, *F*(1, 40) = 3.973, *p* = 0.053. Females in the SH condition showed significantly more freezing than their NE counterparts during time bins 3, 4, 5, 6, 7, and 8 (Figure 5D).

In females exposed to ethanol or water during adolescence, corticosterone levels differed only as a function of stressor, *F*(2, 53) = 160.8, *p* < 0.0001, with no effect of adolescent exposure evident in these animals. Animals in the NE stress condition demonstrated significantly higher corticosterone levels than their HC control counterparts, and the SH group had significantly higher corticosterone levels than both the HC and NE groups (Figure 5E). Similarly, corticosterone levels were not affected by adult exposure and differed only as a function of stressor, *F*(2, 58) = 182.1, *p* < 0.0001, such that females placed in the NE had significantly higher corticosterone levels than the HC control, and animals in the SH group demonstrated greater corticosterone levels than females in both the HC and NE stress conditions (Figure 5F).

**Figure 6:**
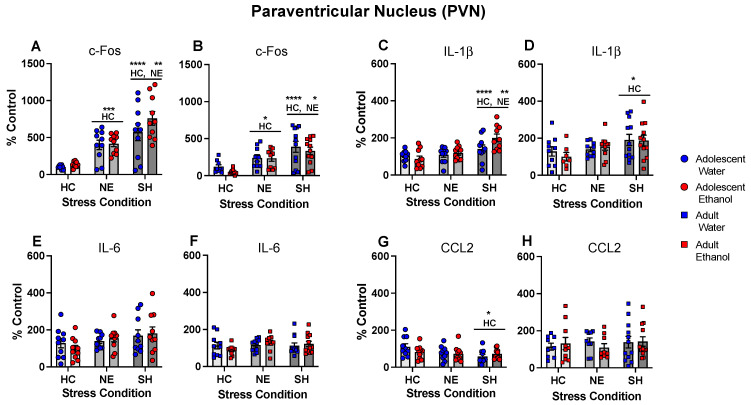
Experiment 4: Gene expression in the paraventricular nucleus (PVN). In both exposure age groups, c-Fos gene expression was enhanced by placement in the NE relative to the HC control, with further enhancement evident under the SH stress condition (**A**,**B**). IL-1β gene expression following adolescent exposure initiation (**C**) was increased in the SH group relative to both the HC and NE conditions, whereas SH increased IL-1β gene expression following adult exposure initiation only relative to the HC control (**D**). IL-6 gene expression was not affected by stressors (**E**,**F**), whereas CCL2 gene expression differed as a function of stress condition following adolescent exposure (**G**) but not adult exposure initiation (**H**), with SH significantly decreasing CCL2 gene expression relative to the HC control. Significant changes relative to the home cage control are marked with HC *—*p* < 0.05, HC **—*p* < 0.01, HC ***—*p* < 0.001, and HC ****—*p* < 0.0001, while significant changes relative to the novel environment stress condition are marked with NE *—*p* < 0.05 and NE **—*p* < 0.01, with data collapsed across exposure.

In the PVN, c-Fos gene expression differed as a function of stress condition following both adolescent, *F*(2, 54) = 63.51, *p* < 0.0001, and adult, *F*(2, 58) = 45.16, *p* < 0.0001, exposure initiation. In both age groups, placement in the NE significantly enhanced c-Fos gene expression relative to HC controls, with further enhancement evident under the SH condition (Figure 6A,B). IL-1β gene expression was affected by stress conditions following adolescent, *F*(2, 54) = 12.08, *p* < 0.0001, as well as adult exposure initiation, *F*(2, 58) = 4.94, *p* < 0.05 (Figure 6C,D), with significant increases in IL-1β gene expression evident only in the SH groups. IL-6 gene expression in the PVN was not affected by stress and exposure conditions following adolescent and adult exposure initiation (Figure 6E,F). CCL2 gene expression differed as a function of stress condition following adolescent, but not adult exposure initiation, *F*(2, 54) = 3.38, *p* < 0.05, with SH significantly decreasing CCL2 gene expression relative to the HC control (Figure 6G,H).

**Figure 7:**
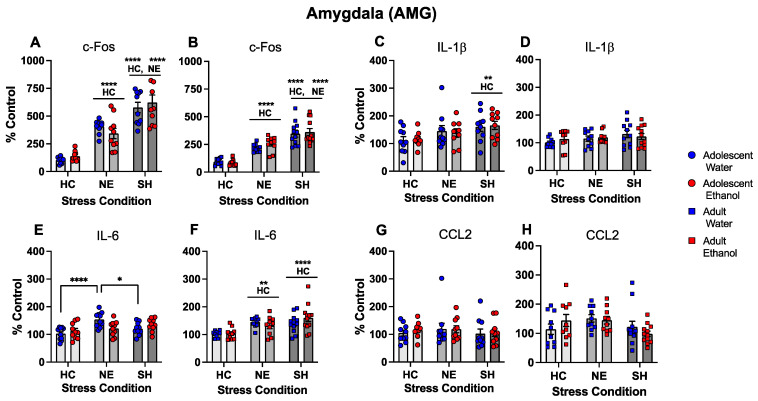
Experiment 4: Gene expression in the amygdala (AMG). In both exposure age groups, c-Fos gene expression (**A**,**B**) was enhanced by placement in the NE relative to the HC control, with further enhancement elicited by SH. IL-1β gene expression following adolescent exposure initiation (**C**) was increased by SH relative to the HC control, with no stress effects evident following adult exposure initiation (**D**). IL-6 gene expression following adolescent exposure initiation (**E**) was significantly increased by placement in the NE in water-exposed females relative to both the HC and SH conditions, with both stress conditions (NE and SH) significantly elevating IL-6 gene expression following adult exposure initiation (**F**). CCL2 gene expression was not affected by stress following adolescent (**G**) or adult (**H**) exposure initiation. Significant changes relative to the home cage control are marked with HC **—*p* < 0.01 and HC ****—*p* < 0.0001, while significant changes relative to the novel environment stress condition are marked with NE ****—*p* < 0.0001, with data collapsed across exposure. Significant stress-associated changes in females exposed to water are marked with *—*p* < 0.05 and ****—*p* < 0.0001.

In the AMG, c-Fos gene expression differed as a function of stress condition following both adolescent and adult exposure initiation, *F*(2, 54) = 72.05, *p* < 0.0001, and *F*(2, 58) = 67.87, *p* < 0.0001, respectively. Placement in the NE significantly enhanced c-Fos gene expression relative to the HC control, with further increases evident in the SH condition that differed significantly from the HC and NE groups (Figure 7A,B). IL-1β gene expression in the AMG was affected by the stress condition only following adolescent exposure initiation, *F*(2, 53) = 4.89, *p* < 0.05, with SH significantly increasing IL-1β gene expression relative to the HC and NE conditions (Figure 7C,D). The two-way ANOVA of IL-6 gene expression following adolescent exposure initiation revealed a significant exposure by stress condition interaction, *F*(2, 54) = 5.18, *p* < 0.01, with the NE placement significantly increasing Il-6 gene expression relative to the HC and SH conditions in water-exposed females, but not in their ethanol-exposed counterparts (see Figure 7E). IL-6 gene expression in the AMG differed as a function of stress condition following adult exposure initiation, *F*(2, 58) = 13.6, *p* < 0.0001, with both stressors significantly elevating IL-6 gene expression relative to the HC control (Figure 7F). CCL2 gene expression in the AMG was not affected by stress and exposure conditions following adolescent and adult exposure initiation (Figure 7G,H).

**Figure 8:**
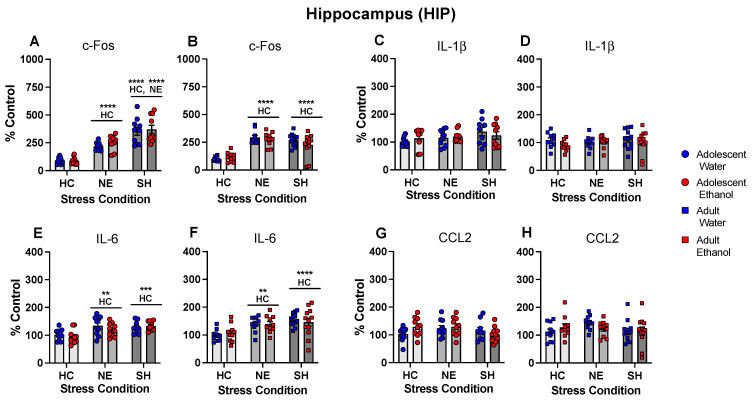
Experiment 4: Gene expression in the hippocampus (HIP). In both exposure age groups, c-Fos gene expression (**A**,**B**) was enhanced by placement in the NE relative to the HC control, with further enhancement evident under the SH condition only following adolescent exposure initiation. IL-1β gene expression in the HPC was not affected by stress (**C**,**D**). IL-6 gene expression was significantly increased by both stressors relative to the HC condition (**E**,**F**). CCL2 gene expression was not affected by stress following adolescent (**G**) or adult (**H**) exposure initiation. Significant changes relative to the home cage control are marked HC **—*p* < 0.01, HC ***—*p* < 0.001, HC ****—*p* < 0.0001, while significant changes relative to the novel environment stress condition are marked with NE ****^—^*p* < 0.0001, with data collapsed across exposure.

In the HPC, c-Fos gene expression differed as a function of stress condition following both adolescent, *F*(2, 54) = 32.57, *p* < 0.0001, and adult, *F*(2, 57) = 16.16, *p* < 0.0001, exposure initiation. Under both initiation conditions, placement in the NE significantly enhanced c-Fos gene expression relative to the HC control, whereas further enhancement was evident under the SH condition only following adolescent exposure initiation (Figure 8A,B). IL-1β (Figure 8C,D) and CCL2 gene expression (Figure 8G,H) in the HPC were not affected by stress and exposure conditions following adolescent and adult exposure initiation. IL-6 gene expression differed as a function of stress condition following both adolescent, *F*(2, 53) = 10.44, *p* < 0.001, and adult, *F*(2, 58) = 12.13, *p* < 0.0001, exposure initiation, with both stressors significantly increasing IL-6 gene expression relative to the HC control following adolescent and adult exposure initiation (Figure 8E,F).

## 5. Discussion

The over-arching goal of this body of work was to test the validity of a novel, consumption-based ethanol exposure procedure in a rat strain typically used for alcohol research. Rats do not typically consume binge level amounts of unsweetened ethanol in voluntary consumption procedures, such as IA2BC or DID paradigms [56,57]. Assessments of ethanol intake as well as blood and brain ethanol concentrations in adult male and female rats (Experiment 1) revealed that BECs and BrECs peaked following access to ethanol for a 2 h period when assessed 1 h into the dark cycle, whereas ethanol intake was substantially higher following longer access periods. The observed decrease in blood ethanol concentration over time may be related to differences in ethanol absorption and elimination rates across the dark part of the cycle. Nutritional states are among the factors that can influence ethanol absorption and elimination in humans, with food in the stomach decreasing BECs and increasing the rate of ethanol metabolism [58]. Similar to human data, the ethanol elimination rate has been reported to be substantially higher in fed rats relative to their food-deprived counterparts [59]. Given that rats are nocturnal, with more than 90% of their food and water intake occurring during the dark part of the cycle [60], it is likely that significant decreases in BECs following access to ethanol for a 6 h period (5 h into the dark cycle) are related to increased ingestion of food, altering the ethanol elimination rate.

Experiment 2 revealed age differences in ethanol intake, blood, and brain ethanol concentration following a 2 h access to ethanol (1 h into the dark cycle), with adolescents ingesting more ethanol and reaching higher BECs as well as BrECs than adults. These findings are in agreement with previously reported age differences in ethanol intake evident under a variety of intake procedures, including both continuous and limited ethanol access [51,52]. Importantly, one third of adolescent animals exposed to a single bottle of ethanol achieved BECs in the binge range (i.e., higher than 80 mg/dL). Although the BECs achieved through the forced consumption model were a fraction of what is typically observed following IG and IP routes of ethanol administration, the range of BECs achieved is, in fact, quite realistic to human binge-like ethanol consumption patterns, strengthening the external validity of this drinking procedure. Although the single-bottle approach does not permit the rat to choose whether to consume ethanol or not, many aspects of the ethanol consumption remain under the rat’s volitional control, since the timing and amount of ethanol consumption, including the level of intoxication they achieve, are self-determined. Importantly, rats continue to gain body weight throughout the ethanol exposure period (see Supplemental Figure S1), even when ethanol consumption occurs during adolescence, a developmental period in which body weight gain and body composition are rapidly changing [18]. In contrast to the proposed model of ethanol exposure, significant decreases in body weight have been reported during adolescent ethanol exposure through vapor inhalation [61] and IP injection [62], as well as during adult exposure via intragastric gavage [63]. Together, these data suggest that intermittent consumption from a single ethanol bottle model is very well tolerated by developing adolescent and young adult males and females. This is consistent with our recent findings with this model, in which rats were exposed to ethanol between 10 and 14 months using this procedure [46]. The ease of implementation, BECs achieved, and ability to implement the procedure easily across vast age ranges suggest that this is a highly tractable model for low-to-moderate ethanol exposure that may have great utility for the field.

To further test the validity of this exposure model, Experiment 3 examined how ethanol consumption would influence neuroimmune reactivity, an effect that we have extensively studied after adolescent ethanol exposure via IG gavage [35,36]. In order to minimize experimenter handling, ethanol consumption was not estimated. Here, we added a novel element by including a comparison between early and late adolescent ethanol exposures, which suggested that ethanol exposure initiated either in early or late adolescence enhanced cytokine expression in response to LPS challenge, an effect that was more evident in females following early ethanol exposure. LPS-induced IL-1β and IL-6 gene expression was enhanced in females exposed to ethanol during early adolescence relative to water-exposed controls challenged with LPS. In contrast, early adolescent exposure through intragastric gavage led to attenuated LPS-induced cytokine gene expression within the cellular fraction of the blood, with females not showing any adolescent exposure-induced alterations in sensitivity to LPS [47]. In follow-up studies, it should be clarified whether this discrepancy between the two studies is related to the mode of ethanol exposure, LPS dose, or challenge timing following the last ethanol exposure. Alternatively, adolescent ethanol exposure might differentially affect central and peripheral LPS-induced cytokine expression. Furthermore, it is important to add more control groups in order to confirm that adolescent ethanol exposure via intermittent ethanol consumption alters responsiveness to LPS, since the only control group injected with a vehicle was not exposed to ethanol during adolescence. These controls will also show whether this novel, consumption-based ethanol exposure alters baseline cytokine genes in the brain or whether this ethanol exposure results in gene expression differences following LPS as a result of neuroimmune priming. A limitation of this experiment is that gene expression changes do not necessarily reflect changes to proteins [64]. Nevertheless, the present findings contribute to a growing body of literature showing that many features of neuroimmune regulation [38,55] and ethanol sensitivity [65] may be sex-dependent.

Overall, there were surprisingly few differences between water and ethanol exposure conditions following adolescent and adult exposure initiation in Experiment 4. Experiment 3 lacked data on ethanol consumption in this paradigm, so Experiment 4 included estimates of ethanol and water consumption within the cage. As expected, more pronounced behavioral and hormonal changes, as well as changes in cytokine gene expression, were evident in the shock condition than following placement in the novel environment, with prior ethanol exposure history not playing a substantial role. IL-1β gene expression was enhanced by shock in the PVN, whereas shock-induced increases in IL-6 gene expression were evident in the hippocampus, suggesting a region-specific neuroimmune response to this stressor. It may be premature to conclude that ethanol exposure at either age does not influence negative affect regulation (fear expression), HPA axis reactivity (Figure 5), or neuroimmune gene expression (Figure 6, Figure 7 and Figure 8) for several reasons. First, Experiment 4 included only female rats, and the duration of the stress challenges was relatively brief (only 30 min). We selected typical footshock procedures in this experiment (or an equivalent exposure to the same novel environment without shock) for two reasons. First, the use of these shock chambers enabled the automated assessment of unconditioned fear responses during and shortly after the shock session. Although no significant interactions between ethanol exposure and stress conditions were observed, there was a trend for adult exposure initiation, with *p* = 0.053, suggesting that adult females may be more sensitive to ethanol than their adolescent counterparts. Additional studies may be required to fully answer this question. Second, shock is a time-honored tradition for which a vast literature examining neural and neuroimmune activation patterns exists [14,66]. Indeed, our recent findings showed that footshock increased IL-1β and several other neuroimmune genes in the PVN of both sexes, though these effects were largely absent among adolescents, possibly indicating that adolescents are less sensitive to the consequences of shock [55]. It is difficult to make a direct comparison between the two studies, since, in Marsland et al. [55], animals received 20 shocks of 5 s each within 30 min, which is substantially more intense than the parameters used here, which were selected because they support the expression of both conditioned and unconditioned fear in both males and females [48]. In another study, we showed that acute ethanol exposure (4 g/kg ig to adult males) led to enhanced HPA axis reactivity, but not IL-1β induction, when a 2 h intermittent session of footshock was imposed during hangover (18 h after 4 g/kg ig) [67]. Regardless, it is notable that, in the present work, adolescents consumed nearly double the amount of ethanol compared to adults yet did not show differential sensitivity to either stress challenge. This may be in part due to faster ethanol clearance rates in adolescents compared to adults, suggesting ethanol intake alone is not the sole determinant of ethanol’s effects on CNS function. A limitation of Experiment 4 is the lack of data on estrous cycle; estrous cycle was not recorded during these experiments, as there were concerns that the vaginal lavage procedure may contribute to an altered history of stress between adolescents and adults. While the estrous cycle does appear to have some effects on fear conditioning and expression, this likely has no effect on the unconditioned fear response [68]. Another limitation of Experiment 4 is that the collection of blood and brain tissue occurred 22 min post-shock; this interval may not be long enough for cytokine gene expression to fully manifest.

## 6. Conclusions

The single-bottle, intermittent consumption model shares certain characteristics with other common AIE exposure models, in which ethanol exposure occurs through IG gavage, IP injection, or vapor inhalation. Such similarities include the age of exposure initiation and the intermittent schedule. Adolescent intermittent exposure to relatively high ethanol doses that bring BECs to 150–200 mg/dL [23,69,70] results in lasting affective alterations that may be related to protracted withdrawal [71]. In the forced consumption model, BECs achieved are relatively low, and future assessment of the long-lasting behavioral consequences associated with this model of ethanol exposure can provide needed information regarding different patterns of drinking in adolescence and adulthood.

## Figures and Tables

**Table 1:** Ethanol intake on Day 1 in Experiments 1 and 2.

Experiment	Experimental Condition	Ethanol Intake (g/kg)	
		Male	Female
Experiment 1	2 h access on Day 2	6.41 ± 2.52	11.54 ± 2.66
	4 h access on Day 2	10.07 ± 2.06	13.15 ± 2.69
	6 h access on Day 2	6.09 ± 1.01	16.87 ± 4.88
Experiment 2	Adolescent	13.34 ± 1.50	14.66 ± 2.64
	Adult	5.22 ± 0.53	7.99 ± 0.70

**Table 2:** Body weights of females exposed to a single bottle of ethanol or water, with exposure initiated either in adolescence or adulthood (Experiment 4).

Cycle	Adolescent Exposure Initiation		Adult Exposure Initiation	
	Water Mean ± SEM (g)	Ethanol Mean ± SEM (g)	Water Mean ± SEM (g)	Ethanol Mean ± SEM (g)
1	109.07 ± 1.78	106.37 ± 1.84	244.91 ± 3.39	238.63 ± 2.87
2	132.27 ± 1.73	127.87 ± 1.92	252.53 ± 3.59	245.50 ± 3.00
3	151.03 ± 1.78	146.80 ± 1.90	257.47 ± 4.47	249.53 ± 3.45
4	165.37 ± 1.74	161.00 ± 1.94	257.69 ± 3.46	249.25 ± 3.37
5	179.00 ± 1.87	172.67 ± 2.00	258.66 ± 3.81	251.78 ± 3.02
6	190.37 ± 2.27	184.60 ± 1.99	262.72 ± 4.06	258.72 ± 3.09
7	201.03 ± 2.27	195.90 ± 1.94	264.03 ± 4.07	261.19 ± 3.75
8	209.33 ± 2.53	207.03 ± 2.11	266.78 ± 3.55	259.67 ± 2.91
9	217.67 ± 2.45	213.20 ± 1.90	267.38 ± 3.52	259.98 ± 3.12
10	225.20 ± 2.81	221.53 ± 1.94	269.78 ± 3.58	262.87 ± 2.92
11	230.80 ± 3.24	225.9 ± 2.01	271.25 ± 3.60	263.53 ± 3.03
12	235.30 ± 30.5	231.23 ± 2.19	271.50 ± 3.56	263.59 ± 3.00

## Data Availability

The data presented in this study are available on request from the corresponding author.

## Supplementary Materials

The following are available online at https://www.mdpi.com/article/10.3390/cells12151991/s1, Figure S1: Body weight during consumption period.
