# Supplementary material for: Intermittent Exposure to a Single Bottle of Ethanol Modulates Stress Sensitivity: Impact of Age at Exposure Initiation
Source: Cells. 2023 Aug 3;12(15):1991. doi: 10.3390/cells12151991 (PMC10417636; doi:10.3390/cells12151991)
Supplement: Supplementary file 1 [file cells-12-01991-s001.zip › cells-2489232-supplementary.pdf]

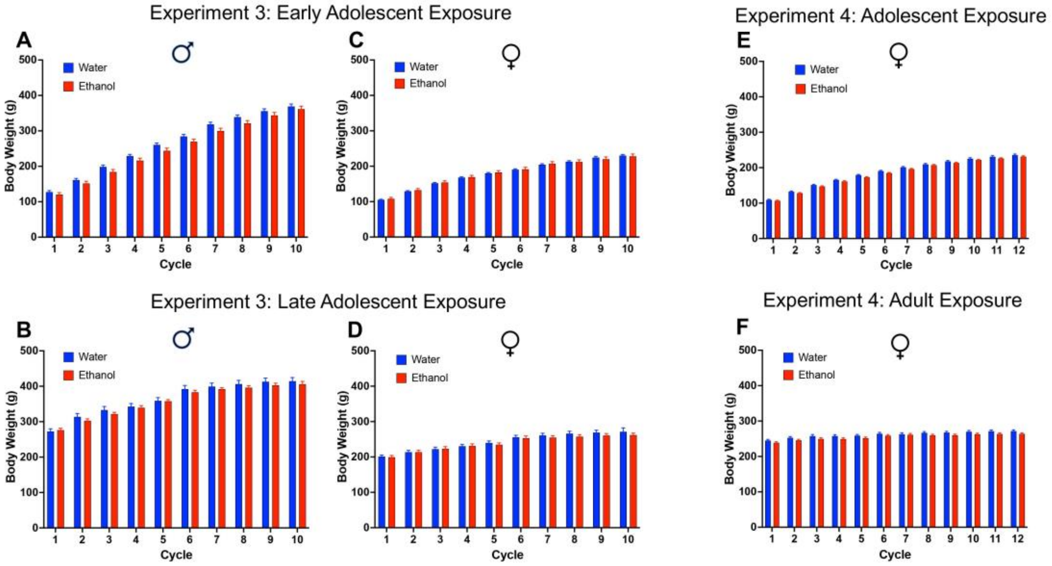

Figure S1. Supplemental Figure 1. Body weight changes across exposure cycles in Experiments 3 and 4. In Experiment 3, intermittent exposure to a single bottle with ethanol did not affect body weights of males during both early (A) and late (B) adolescent exposures. Body weight of adolescent females were not affected by ingested ethanol across early (C) and late (D) exposures. In Experiment 4, neither adolescent (E) nor adult ethanol exposure (F) affected body weight. All experimental subjects showed gradual and age-appropriate increases in body weight.
